# Gut Microbiota-Mediated Histidine Deficiency Drives Testicular Ferroptosis Induced by Bisphenol F Exposure

**DOI:** 10.3390/antiox15060714

**Published:** 2026-06-04

**Authors:** Bo-Yang Zhang, Yue-Qi Wang, Rui Yang, Yan Zhang, Dao-Zhen Jiang, Li-Hong Ji, Yi-Fei Mao, Bo Tang, Xue-Ming Zhang

**Affiliations:** 1 College of Veterinary Medicine, Jilin University, 5333 Xi-An Road, Changchun 130062, China; zby23@mails.jlu.edu.cn (B.-Y.Z.); yueqiw22@mails.jlu.edu.cn (Y.-Q.W.); jiangdz23@mails.jlu.edu.cn (D.-Z.J.); jilh9923@mails.jlu.edu.cn (L.-H.J.); maoyf24@mails.jlu.edu.cn (Y.-F.M.); tang_bo@jlu.edu.cn (B.T.); 2 College of Veterinary Medicine, Gansu Agricultural University, Lanzhou 730070, China; ruiyang22@mails.jlu.edu.cn

**Keywords:** endocrine-disrupting chemical, bisphenol F, ferroptosis, gut microbiota, histidine metabolism, testes

## Abstract

Bisphenol F (BPF), a widespread environmental contaminant and a major substitute for the restricted bisphenol A (BPA), has raised increasing concerns regarding its potential male reproductive health risks, yet its underlying mechanisms remain poorly understood. This study investigates the mechanisms underlying BPF-induced testicular damage, focusing on the interplay among gut microbiota (GM) dysbiosis, histidine metabolism disruption, and ferroptosis. Using a mouse model exposed to BPF (50, 100, and 200 mg/kg/day) for 28 days, we observed significant testicular pathology, including seminiferous tubule atrophy, vacuolation, and blood-testis barrier (BTB) impairment. Metagenomic and metabolomic analyses revealed GM dysbiosis and suppressed intestinal histidine metabolism, accompanied by decreased abundance of beneficial taxa (e.g., *Bacteroides*, *Ligilactobacillus*) and increased potential pathobionts (e.g., *Akkermansia*, *Mucispirillum*). BPF exposure was associated with reduced testicular histidine levels and decreased expression of the histidine transporter-related marker LAT1, suggesting impaired histidine availability and a possible alteration in LAT1/CD98-mediated transport; however, direct inhibition of LAT1/CD98 transport activity was not experimentally demonstrated. BPF exposure was accompanied by ferroptosis-related alterations in the testes, including mitochondrial damage, iron accumulation, lipid peroxidation, and downregulation of the xCT-GSH-GPX4 antioxidant axis. In vitro experiments using mouse Sertoli cells (mSCs) confirmed BPF-induced ferroptosis, which was mitigated by the exogenous histidine supplementation. Histidine administration in vivo ameliorated testicular damage, restored BTB integrity, and reversed ferroptotic markers. Our findings support a working model in which a GM–histidine–testis axis may contribute to BPF-induced reproductive toxicity, while further functional studies are required to establish direct causality and transporter-level mechanisms.

## 1. Introduction

In recent decades, male infertility has emerged as a significant global health concern, with studies reporting a dramatic decline in sperm quality and quantity. A comprehensive meta-regression analysis revealed that sperm counts have dropped by nearly 60% over the past 40 years, highlighting an alarming trend that underscores the role of environmental factors in reproductive health. Among these factors, endocrine-disrupting chemicals (EDCs) have garnered considerable attention due to their pervasive presence and potential to interfere with hormonal systems [1]. Bisphenol A (BPA), a well-known EDC used in plastics and epoxy resins, has been extensively studied and linked to adverse reproductive outcomes [2]. In response to regulatory restrictions on BPA, structural analogs such as Bisphenol F (BPF) have been increasingly adopted in industrial applications [3].

Despite its widespread use, BPF has also been associated with carcinogenic and reproductive toxicities, raising concerns about its safety as a BPA substitute [4]. While neurotoxic effects of BPF have been documented, its impact on the male reproductive system—particularly the testes—remains inadequately characterized. The testes are highly susceptible to environmental toxicants, which can disrupt spermatogenesis, impair the blood-testis barrier (BTB), and induce oxidative stress, ultimately leading to infertility [5].

Recent evidence suggests that BPF may exert its toxicity not only through direct cell/tissue damage but also via indirect pathways involving the gut microbiota (GM) and metabolic homeostasis. The gut-testis axis has emerged as a critical interface between environmental exposures and reproductive health, wherein dysbiosis and metabolic alterations can amplify toxicological responses [6].

Furthermore, ferroptosis—a form of regulated cell death driven by iron-dependent lipid peroxidation—has recently been implicated in chemical-induced testicular injury. Several environmental toxicants, including cadmium and microplastics, have been reported to trigger ferroptosis in testicular cells, leading to BTB dysfunction and spermatogenic failure [7,8]. Whether BPF induces testicular damage through a similar ferroptotic pathway remains unclear.

This study aims to systematically decipher the mechanism of BPF-induced testicular injury by integrating multi-omics and functional validation. We hypothesized that BPF exposure may disrupt the gut microbiota and histidine metabolism, thereby reducing histidine availability in the testis and increasing susceptibility to ferroptosis-related injury through impairment of the xCT-GPX4 antioxidant axis. Our findings not only elucidate a novel toxicological pathway for an emerging environmental contaminant but also provide mechanistic evidence for assessing its male reproductive health risks and developing targeted mitigation strategies.

## 2. Materials and Methods

### 2.1. Materials

BPF (B806539) were bought from Macklin (Shanghai, China). Antibodies against Claudin 1 (13,050–1-AP), ZO-1 (21,773–1-AP), and β-actin (66,009–1-lg) were purchased from Proteintech (Wuhan, China). Antibodies for xCT (T56957) and GPX4 (T56757) were bought from Abmart (Shanghai, China). The culture medium and ingredients were sourced from Procell (Wuhan, China).

### 2.2. BPF-Exposed Mouse Model

Male Kunming mice (7–8 weeks old) provided by the Experimental Animal Center of Bethune Medical College at Jilin University were maintained under standard laboratory conditions (22–23 °C, 12-h light/dark cycle) with free access to food and water. This study was approved by the Institutional Animal Care and Use Committee of Jilin University and was strictly conducted in compliance with the revised ARRIVE 2.0 guidelines. Based on preliminary experiments, BPF was first dissolved in 100% dimethyl sulfoxide (DMSO, Sigma, Kawasaki, Japan, D4540) to prepare a stock solution. The stock solution was diluted with corn oil for final oral gavage. The final DMSO concentration in the gavage solution was 5% (*v*/*v*) for all groups, and the gavage volume was 200 μL per 20 g mouse (10 mL/kg body weight). The animals were randomly allocated into four groups (n = 6/group): vehicle control (Con, receiving an equal volume of 5% DMSO in corn oil without BPF), BPF-50 (50 mg/kg/day), BPF-100 (100 mg/kg/day), and BPF-200 (200 mg/kg/day). All treatments were administered via oral gavage once daily for 28 consecutive days. Randomization: Mice were randomly assigned to four groups using a computer-generated random number table. Blinding: The investigator performing oral gavage was not blinded, but all outcome assessments (histopathology, immunofluorescence, Western blot, TEM) were conducted by researchers blinded to group allocation. Codes were broken after data collection. After the treatment period, all mice were euthanized by cervical dislocation, the testes and serum samples were collected for subsequent histopathology, immunofluorescence, and ELISA analyses. Humane endpoints were predefined in this study. Mice were observed twice daily (morning and afternoon) during the experiment. Monitored signs included: rapid weight loss (>20% loss in a single day or progressive decline), piloerection, hunched posture, lethargy, respiratory distress, and reduced food/water intake. Mice were immediately euthanized if they exhibited >25% body weight loss, inability to right themselves, severe respiratory distress, or persistent convulsions.

### 2.3. Histopathological Examination

The testes were embedded in paraffin wax after fixation in 4% formaldehyde solution for 24 h to create 5 μm-thick slices. In order to observe the histopathological changes of the testicular tissues, the sections were stained with hematoxylin-eosin (HE) regularly, observed, and photographed under a light microscope. As described previously [9], based on the integrity of the testicular seminiferous epithelium and the degree of vacuolization in the seminiferous tubules, three experienced histopathology analysts randomly selected three fields of view from paraffin sections for scoring (0, no damage; 1, mild damage; 2, moderate damage; 3, severe damage; 4, very acute damage).

### 2.4. Immunofluorescence

Immunofluorescent staining of the testicular tissues was carried out as we described before [9]. Briefly, the paraffin sections were deparaffinized, rehydrated, and further treated with ethylene diamine tetraacetic acid antigen retrieval buffer (pH 8.0). Then the sections were blocked with 3% bovine serum albumin (BSA, *w*/*v*) for 30 min. Subsequently, they were incubated overnight at 4 °C with rabbit anti-ZO-1 and rabbit anti-Claudin-1 (1:200, respectively). Next, the sections were incubated with Alexa Fluor 488-conjugated goat anti-rabbit IgG (S0018, 1:200, Affinity Biosciences, Changzhou, China) for 60 min. To visualize the nuclei, the samples were counterstained with 4′, 6-diamidino-2-phenylindole (DAPI, Beyotim Biotechnology, Shanghai, China) and incubated at room temperature for 15 min. Finally, the sections were covered with anti-quenching tablets and observed under a Nikon 80i fluorescence microscope (Tokyo, Japan). The sections stained with isotype IgG served as the negative control. For the fluorescence intensity analysis, at least three visual fields were selected randomly in each group.

### 2.5. Western Blot

To obtain the total protein, mouse testicular tissues and the mouse Sertoli cells (mSCs) were lysed with lysis buffer (Beyotime, Shanghai, China), followed by centrifugation for 15 min at 12,000× *g*. The supernatants containing the total protein were collected. Subsequently, the protein concentrations were determined by using the BCA Protein Assay Kit (ThermoFisher Scientific, Waltham, MA, USA) according to the manufacturer’s instructions. Protein samples were loaded onto 6–15% SDS-PAGE at 110 V for 90 min and then transferred onto a polyvinylidene difluoride membrane (Millipore, Darmstadt, Germany) at 75 V for 1 h. The transferred membranes were blocked in 0.1% Tris buffered saline-Tween (TBS-T) containing 5% skim milk for 2 h, followed by incubation overnight at 4 °C with each primary antibody (xCT, 1:1000; GPX4, 1:1000; ZO-1, 1:1000; β-actin, 1:2000) in TBS-T containing 5% BSA (Gentihold, Beijing, China). After washing 5 times with TBS-T, the membranes were co-incubated with the corresponding horseradish peroxidase-labeled secondary antibodies (1:3000, Bosterbio, Pleasanton, CA, USA) at room temperature for 1 h. Finally, the immunoreactive blots were visualized with an enhanced chemiluminescent substrate (ThermoFisher Scientific, Waltham, MA, USA).

### 2.6. Quantitative Real-Time Reverse Transcription-PCR (qRT-PCR)

Total RNAs were isolated from the mouse feces and testicular tissues by using the Trace RNA Extraction Kit (Invitrogen, Carlsbad, CA, USA) and then reverse-transcripted into cDNAs by using the Revert Aid First Strand cDNA Synthesis Kit (ThermoFisher Scientific, Waltham, MA, USA) according to the manufacturer’s instructions. The qRT-PCR was performed with the PerfectStart^®^ Green qPCR SuperMix (TransGen Biotech, Beijing, China) following the manufacturer’s instructions. The primers were designed by Sangon Biotech (Shanghai, China), and the sequences are listed in Table S1. 16s was used as an internal reference gene, and the relative mRNA levels of the target genes were calculated by the 2^−ΔΔCt^ method.

### 2.7. Cell Culture and Treatment

The mouse Sertoli cell line (mSCs, TM4, Procell CL-0456) was obtained from Procell Life Science & Technology Co., Ltd. (Wuhan, China) Cells were cultured in Dulbecco’s Modified Eagle Medium/Nutrient Mixture F-12 (DMEM/F12, PM150312) supplemented with 5% Hartmann’s solution (164215-500), 2.5% fetal bovine serum (FBS, 164210-500), and 1% penicillin-streptomycin (PB180120). The cells were maintained at 37 °C in a humidified incubator with 5% CO_2_. For in vitro cell experiments, mSCs were harvested and seeded into appropriate culture plates (6-well, 96-well, etc.) upon reaching 80–90% confluence and allowed to adhere overnight. The following treatment paradigms were employed.

### 2.8. BPF Cytotoxicity and Dose-Response

To determine the cytotoxic effects of BPF, mSCs were treated with a range of BPF concentrations (6.25, 12.5, 25, 50, 100, 200, and 400 μM) for 24 h. Based on the results, concentrations of 25, 50, and 100 μM (which induced approximately 50% cell mortality) were selected for subsequent experiments to investigate ferroptosis mechanisms. Cells were treated with these concentrations for 24 h before sample collection for analyses, including malondialdehyde (MDA S0131S Beyotime, Shanghai, China) and glutathione (GSH S0053 Beyotime, Shanghai, China) assays, Western blot, intracellular Fe^2+^, reactive oxygen species (ROS S0033S Beyotime, Shanghai, China), and transmission electron microscopy (TEM).

### 2.9. Inhibition of Ferroptosis

To confirm the role of ferroptosis in BPF-induced toxicity, the mSCs were pre-treated with the specific ferroptosis inhibitor Ferrostatin-1 (Fer-1, 1 μM HY-100579, MedChemExpress, Monmouth Junction, NJ, USA) for 2 h, followed by co-exposure with BPF (100 μM) for an additional 24 h. Cells were then collected for assessment of intracellular Fe^2+^, ROS, cell viability, MDA, GSH, and protein expression.

### 2.10. Histidine Supplementation Rescue

To investigate the protective role of histidine against BPF-induced ferroptosis, mSCs were treated with BPF (100 μM) alone or in combination with exogenous L-Histidine (H8060, Macklin, Shanghai, China) at various concentrations (1, 2, and 5 μM) for 24 h. Following treatment, cells were harvested to evaluate ferroptosis-related parameters, including Fe^2+^ accumulation, ROS levels, cell viability, MDA and GSH content, and the expression of xCT and GPX4 proteins.

### 2.11. Shotgun Metagenomic Sequencing and Analysis

The cecum fecal samples (100 mg/mouse) were collected under sterile conditions from the animals in the BPF-200 group (200 mg/kg/day for 28 d) and from the control group (n = 6 per group), and stored at −80 °C. Shotgun metagenomic sequencing and analysis were prepared as previously described and supported by Annoroad Gene Technology Co., Ltd. (Beijing, China). Genomic DNA was extracted using a QIAGEN kit and monitored by electrophoresis on a 1% agarose gel. The quality of the DNA samples was further quantified using a Qubit^®^ 2.0 fluorometer (Life Technologies, Carlsbad, CA, USA) with an OD value between 1.8 and 2.0. For library construction, a total of 1 μg of DNA per sample was used as input material for the DNA sample preparations. Sequencing libraries were generated using the NEBNext^®^ UltraTM DNA Library Prep Kit for Illumina (NEB, Ipswich, MA, USA) following the manufacturer’s recommendations, and index codes were added to attribute sequences to each sample. Briefly, the DNA sample was fragmented by sonication to a size of 350 bp. The DNA fragments were end-polished, A-tailed, and ligated with the full-length adaptor for Illumina sequencing with further PCR amplification. Finally, PCR products were purified (AMPure XP system), and libraries were analyzed for size distribution by using an Agilent2100 Bioanalyzer (Santa Clara, CA, USA) and were quantified using real-time PCR. After the index-coded sample clusters were generated on a cBot Cluster Generation System according to the manufacturer’s instructions, the library preparations were sequenced on an Illumina NovaSeq platform (San Diego, CA,USA), and paired-end reads were generated, with at least 6 Gb reads per sample.

We used Readfq (V8, https://github.com, accessed on 7 April 2025) to acquire the clean data for subsequent analysis. Host sequences were then discarded by mapping the sequences against the reference genome (hg19) using BowTie2.2.4 (http://bowtie-bio.sourceforge.net/bowtie2/index.shtml, accessed on 7 April 2025). The remaining set of clean reads was pooled and subjected to metagenomics by using SOAP- denovo software (V2.04, http://soapdenovo2.sourceforge.net/, accessed om 7 April 2025). The assembled scaftigs were broken at sites containing ‘N’ bases, and only segments without Ns were retained. The clean reads from all samples were then mapped back to these scaffolds using Bowtie2.2.4 to identify the paired-end (PE) reads that had not been incorporated into the assembly. The assembled scaftigs (>500 bp) were predicted as ORFs by MetaGeneMark (V2.10, http://topaz.gatech.edu/GeneMark/, accessed on 10 April 2025) software, and the length information shorter than 100 nt was filtered. CD-HIT software (V4.5.8, https://github.com/weizhongli/cdhit, accessed on 10 April 2025) was adopted for redundancy and to obtain the unique initial gene catalog and ORF prediction. DIAMOND software (V0.9.9, https://github.com/bbuchfink/diamond/, accessed on 10 April 2025) was used to BLAST the unigenes against the sequences of bacteria, fungi, archaea, and viruses, which were all extracted from the NR database (Version: 2018–01-02, https://www.ncbi.nlm.nih.gov/, accessed on 10 April 2025). We used the LCA algorithm, which is applied to the system classification of MEGAN software(version 6.12.3, built 14 Aug 2018), to ensure the species annotation information of sequences. We adopted DIAMOND software (V0.9.9) to BLAST unigenes to the functional database. The functional database includes the KEGG database (Version 2018–01-01, http://www.kegg.jp, accessed on 10 April 2025), eggNOG database (Version 4.5, http://eggnog.embl.de, accessed on 10 April 2025), and CAZy database (Version 201,801, http://www.cazy.org/, accessed on 10 April 2025). For each sequence’s BLAST result, the best BLAST hit was used for subsequent analysis. For differential abundance analysis of microbial taxa, the Benjamini-Hochberg FDR correction was applied, with a significance threshold of q < 0.05.

### 2.12. Non-Targeted Metabolic Profiling

Cecal fecal samples from 6 mice per group (control and BPF-200) were frozen with liquid nitrogen, ground individually, and the homogenate was resuspended with prechilled 80% methanol by vortexing. The samples were incubated on ice for 5 min and then centrifuged at 15,000× *g*, 4 °C for 20 min. Some of supernatant was diluted to final concentration containing 53% methanol by LC-MS grade water. The samples were subsequently transferred to a fresh Eppendorf tube and then centrifuged at 15,000× *g*, 4 °C for 20 min. Finally, the supernatant was injected into the LC-MS/MS system for analysis [10].

UHPLC-MS/MS analyses were conducted using a Vanquish UHPLC system (Thermo Fisher, Dreieich, Germany) coupled with either an Orbitrap Q Exactive™ HF mass spectrometer, Orbitrap Q Exactive™ HF-X mass spectrometer, or Orbitrap Exploris™ 120 mass spectrometer (Thermo Fisher, Germany) at Novogene Co., Ltd. (Beijing, China). Two chromatographic methods were employed: Method ① utilized a Hypersil Gold column (100 × 2.1 mm, 1.9 μm) with a 12-min linear gradient at 0.2 mL/min flow rate, using eluent A (0.1% formic acid in water) and eluent B (methanol) for both positive and negative polarity modes. The gradient program was: 2% B at 1.5 min, 2–85% B at 3 min, 85–100% B at 10 min, 100–2% B at 10.1 min, and 2% B at 12 min. Method ② employed an ACQUITY UPLC BEH Amide column (100 × 2.1 mm, 1.7 μm) with the same flow rate and duration, using eluent A (5 mM ammonium acetate in 90% acetonitrile) and eluent B (5 mM ammonium acetate in 50% acetonitrile). The gradient for Method ② was: 2% B at 1.5 min, 2–100% B at 7 min, 100% B at 9 min, 100–2% B at 9.1 min, and 2% B at 12 min. Mass spectrometry parameters were maintained at spray voltage of 3.5 kV, capillary temperature of 320 °C, sheath gas flow rate of 35 psi (or arb), auxiliary gas flow rate of 10 L/min (or arb), S-lens RF level of 60, and auxiliary gas heater temperature of 350 °C for both polarity modes.

The raw data files from UHPLC-MS/MS were processed using XCMS for peak alignment, peak picking, and metabolite quantification. Metabolite identification was achieved by comparing the data against the self-built high-quality secondary spectrum database (NovoMetDB) with a mass deviation setting of 10 ppm, considering adduct ions. Background ions identified from blank samples were removed, and the original quantitative data were normalized using the formula: Relative peak areas = Raw quantitative value of samples/(Sum of quantitative values of samples/Sum of quantitative values of QC1). Metabolites with a coefficient of variation (CV) greater than 30% in QC samples were excluded. All data processing was performed on a Linux operating system (CentOS version 6.6) using R (3.2.X) and Python (2.6.6), with specific package and software details provided in the README file.

Metabolites were annotated using the KEGG (Kyoto Encyclopedia of Genes and Genomes), HMDB (Human Metabolome Database), and LIPIDMaps databases. Multivariate statistical analyses, including principal component analysis (PCA) and partial least squares discriminant analysis (PLS-DA), were conducted using metaX software (1.0.3). Univariate analysis (*t*-test) was applied to calculate statistical significance (*p*-value), and fold change (FC) was determined as the ratio of the mean quantitative values between comparison groups. Volcano plots (using ggplot2 in R) based on log2 (FoldChange) and −log10 (*p*-value), and clustering heatmaps (using Pheatmap in R) with z-score normalization of intensity areas were included for visualization. Correlation analysis between differential metabolites was performed using Pearson’s method cor () in R, with statistical significance assessed by cor.mtest () (*p*-value < 0.05) and visualized using corrplot. Functional enrichment of metabolic pathways was evaluated using the KEGG database; pathways were considered enriched when the ratio x/n exceeded y/N, and statistically significant when *p*-value < 0.05. Differentially abundant metabolites between groups were identified based on a combination of fold change (FC > 1.5 or <0.667) and unpaired Student’s *t*-test (*p* < 0.05). No formal false discovery rate (FDR) correction was applied to the metabolomics data.

### 2.13. Fe^2+^ Immunostaining in mSCs

Intracellular ferrous iron (Fe^2+^) levels in mSCs were detected using the FeRhoNox™-1 probe (SCT030 Goryo Chemical, Sapporo, Japan) according to the manufacturer’s protocol. Briefly, after the respective treatments (e.g., BPF, Ferrostatin-1, and/or L-Histidine), cells cultured in 96-well plates or on coverslips were washed twice with pre-warmed Hanks’ Balanced Salt Solution (HBSS). The cells were then incubated with a working solution of FeRhoNox™-1 (5 μM) in HBSS at 37 °C for 30 min in the dark. Following incubation, the staining solution was removed, and cells were gently washed three times with pre-warmed HBSS to remove excess probe. For fluorescence microscopic observation, cells on coverslips were mounted and immediately visualized under a fluorescence microscope. Three random fields per group were captured for mean fluorescence intensity analysis using ImageJ software (1.54m).

### 2.14. ROS Assay

Intracellular ROS levels were detected using the Reactive Oxygen Species Assay Kit (Beyotime, S0033S, Shanghai, China). After treatments, mSCs on coverslips were washed with PBS and loaded with 10 μM DCFH-DA in serum-free medium at 37 °C for 30 min in the dark. Following PBS washes, the cells were immediately observed under a fluorescence microscope. Three random fields per group were captured for mean fluorescence intensity analysis using ImageJ software.

### 2.15. Metabolite Treatment

L-Histidine was dissolved in physiological saline. Twenty-four BPF-exposed mice (200 mg/kg/day for 28 d) were randomly assigned to 4 groups (n = 6/group). Histidine was administered via oral gavage at doses of 0, 25, 50, and 100 mg/kg once daily for 4 consecutive weeks. Non-treated mice (n = 6) were used as the control. Following the treatment period, all animals were euthanized by cervical dislocation under deep anesthesia with isoflurane, and the testicular tissues, along with serum samples, were collected for subsequent HE, immunostaining of ZO-1 and Claudin-1, MDA, and GSH analyses.

### 2.16. Transmission Electron Microscopy (TEM)

TEM was performed for mitochondrial observation as previously described. Briefly, the testicular tissues and cultured mSCs were fixed with 2.5% glutaraldehyde and post-fixed in 1% osmium tetroxide. The prepared samples were dehydrated in a graded series of ethanol and acetone. Next, the samples were embedded in Epon resin (Electron Microscopy Sciences, Hatfield, PA, USA). The ultrathin sections were created and stained with lead citrate and uranyl acetate. Electron micrographs were analyzed with a JEM-1400 Plus transmission electron microscope (JEOL Ltd., Tokyo, Japan). In the testicular tissues, Sertoli cells were observed mainly.

### 2.17. Cell Viability Assay

The Cell Proliferation and Cytotoxicity Assay Kit (Cell Counting Kit-8, CCK-8, CA1210, Solarbio, Beijing, China) was employed to investigate the cytotoxicity of BPF on mSCs. Briefly, mSCs were treated with BPF at 6.25, 12.5, 25, 50, 100, 200, and 400 μM for 24 h, respectively. Subsequently, the cells were treated with BPF (25, 50, 100 μM) for 24 h according to the manufacturer’s instructions, and the samples were collected for MDA and GSH assay. For Fer-1 and histidine treatment, mSCs were initially incubated with 100 μM BPF.

### 2.18. ELISA

The testicular tissues, serum, and fecal samples were prepared for ELISA assay. Histidine ELISA Kit and BPF ELISA Kit (both from Jiangsu Meimian Industrial Co., Ltd., Yancheng, China) detections were performed according to the instructions. The absorbance of each well was measured at a wavelength of 450 nm within 15 min.

### 2.19. Iron Assay

Tissue iron concentrations were measured using Iron Assay Kit (S0161S, Beyotime, Shanghai, China) according to the manufacturer’s instructions. In brief, testicular tissue (10 mg) was rapidly homogenized in 6 × Iron Assay buffer and centrifuged at 16,000× *g* for 10 min at 4 °C to remove insoluble material. Samples (50 μL in each group) were added into 96-well plate, and the volumes were brought to 100 μL with 50 μL Assay buffer. Next, 5 μL of Iron assay buffer was added for ferrous iron, respectively. After mixing and incubating in the dark for 30 min at 25 °C, 100 μL Iron Probe was added to each well and incubated in the dark for 60 min at 25 °C. Finally, the absorbance was measured at 593 nm, and the standard curve line was used for iron concentration calculation.

### 2.20. Malondialdehyde (MDA) and Glutathione (GSH) Assay

Testicular tissue (10 mg) and mSCs (1 × 106 cells) were homogenized on ice in 300 μL MDA Lysis Buffer (P0013 Beyotime, Shanghai, China) containing 3 μL BHT (100×) and centrifuged at 13,000× *g* for 10 min to remove insoluble material. The MDA content was measured using Lipid Peroxidation Assay Kit according to the manufacturer’s instructions (Beyotime, S0131S, Shanghai, China). The testicular tissue and mSCs were also collected to measure GSH levels according to the manufacturer’s instructions (A006-2, Nanjing Jiancheng Bioengineering Institute, Nanjing, China).

### 2.21. Statistical Analysis

The experiments were all carried out in triplicate. Data were presented as mean ± SEM and analyzed by GraphPad Prism 10 (La Jolla, CA, USA). Differences between groups were analyzed by parametric one-way analysis of variance (ANOVA), and pairwise comparisons were performed by least-significant difference. To address concerns about multiple comparisons, we verified all significant findings using Tukey’s HSD post-hoc test. All reported primary endpoint differences remained significant, confirming the robustness of our conclusions. *p* < 0.05 is considered statistically significant (* *p* < 0.05, ** *p* < 0.01, *** *p* < 0.001, **** *p* < 0.0001).

## 3. Results

### 3.1. BPF Causes Testicular Damage

**Figure 1:**
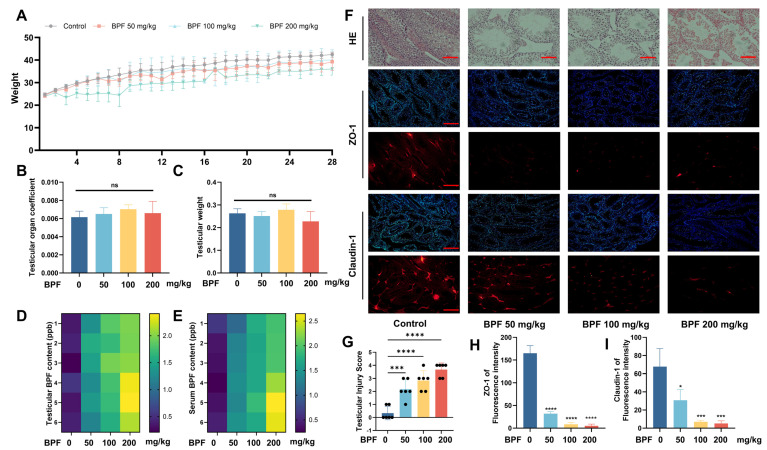
BPF causes testicular damage. Mice were pre-treated with BPF (0, 50, 100, and 200 mg/kg/day respectively) for 28 d. (**A**) Body weight growth curves showing dramatic deceleration in BPF-treated groups. (**B**) Final testicular weights at endpoint measurement; (**C**) Testicular organ indices (testis/body weight ratio); (**D**) BPF levels in the testicular tissues; (**E**) BPF levels in the serum samples; (**F**) Hematoxylin and eosin (HE) staining showing seminiferous epithelial damage, scale bar = 50 μm. Immunofluorescence of ZO-1 and Claudin-1 (red) with DAPI counterstain (blue), demonstrating disrupted BTB integrity. Scale bar = 100 μm. (**G**) Histopathological score of testicular tissue; (**H**,**I**) Fluorescence intensity of ZO-1 and Claudin-1 according to panel (**F**). ns = *p* > 0.05, * *p* < 0.05, *** *p* < 0.001, **** *p* < 0.0001. Data are mean ± SEM; n = 6 per group; one-way ANOVA with Tukey’s post hoc test.

Firstly, a BPF-exposed mouse model was developed, and the changes in body weight were recorded over 28 days. The results showed a dose-dependent deceleration in body weight gain, but no statistical changes in the testicular weights were observed (Figure 1A–C). Subsequently, to investigate how BPF enters testicular tissue, we measured BPF levels in both testicular tissues and serum. The analysis revealed that elevated BPF concentrations were observed in both compartments following BPF exposure (Figure 1D,E). Furthermore, histopathological examination revealed severe structural damage to the seminiferous epithelium in all BPF-treated groups compared to the control, characterized by atrophy and loosening of the seminiferous epithelium, and extensive vacuolation (Figure 1F,G). Given that ZO-1 and Claudin-1 are core structural proteins of the blood-testis barrier (BTB), we performed immunofluorescence staining for both proteins in the testicular tissues. The decreased expressions of ZO-1 and Claudin-1 in BPF-treated groups (Figure 1F,H,I) indicate that BPF can disrupt the BTB integrity. These findings demonstrate that BPF can enter testes through the circulatory system and induce testicular damage.

### 3.2. BPF Induces Abnormal Histidine Metabolism

**Figure 2:**
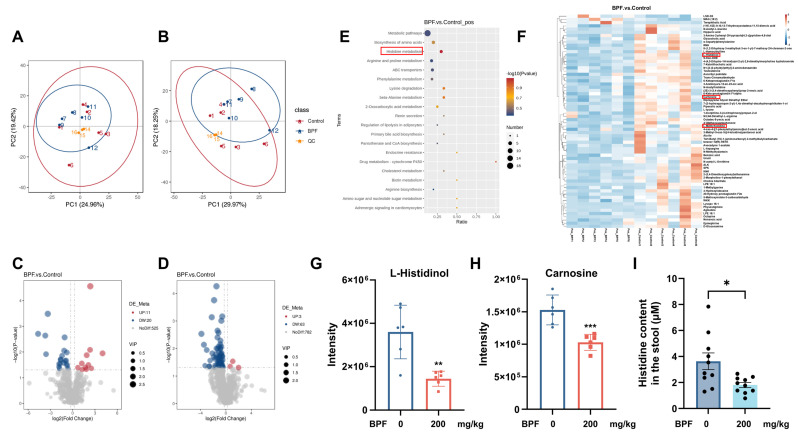
BPF causes intestinal metabolic disorders in mice. (**A**,**B**) Principal component analysis (PCA) showing distinct clustering of intestinal metabolites between the BPF-200 and Control groups; (**C**,**D**) Volcano plot analysis identifying 52 upregulated and 32 downregulated metabolites; (**E**,**F**) KEGG enrichment analysis revealing significant suppression of the histidine metabolism pathway in BPF-treated mice; (**G**,**H**) Reduced levels of histidine synthesis precursor (L-Histidinol) and histidine-related metabolite (Carnosine) after BPF exposure; (**I**) ELISA validation confirming decreased histidine levels in the feces of BPF-exposed mice. * *p* < 0.05, ** *p* < 0.01, *** *p* < 0.001. Data are mean ± SEM; n = 6 per group; *t*-test for (**G**–**I**).

**Figure 3:**
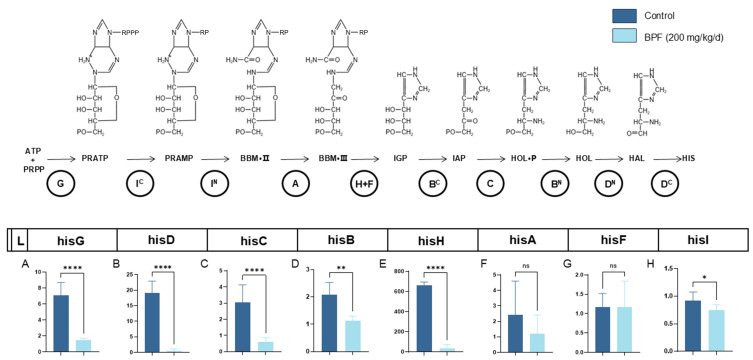
BPF reduces the enzyme expression in the intestinal histidine metabolism pathway. (**A**–**H**) qRT-PCR analysis confirmed the downregulated expressions of 8 key enzymes in the histidine metabolic pathway. The enzymes encoded by the relative cistrons are indicated in the circles below the arrows. When a gene encodes a bifunctional enzyme, the domains performing the single reaction, amino or carboxyl terminal, are indicated by a superscript N or C, respectively. ns = *p* > 0.05, * *p* < 0.05, ** *p* < 0.01, **** *p* < 0.0001. RPPP (ribosyl triphosphate); RP (ribosyl phosphate); PRPP (5-phosphoribosyl-1-pyrophosphate); PRATP (N′-5′-phosphoribosyl-ATP); PRAMP (N′-5′-phosphoribosyl-AMP); BBM-II (5′-ProFAR, N′-[(5′-phosphoribosyl)-formimino]-5-aminoimidazole-4-carboxamide ribonucleotide); BBM-III (5′-PRFAR, N′-[(5′-phosphoribulosyl)-formimino]-5-aminoimidazole-4-carboxamide ribonucleotide); IGP (imidazole glycerol phosphate); ZMP (AICAR, 5′-phosphoribosyl-4-carboxamide-5-aminoimidazole); IAP (imidazole acetol phosphate); HOL-P (L-histidinol phosphate); HOL (L-histidinol); HAL (L-histidinal); HIS (L-histidine). Data are mean ± SEM; n = 6 per group; *t*-test.

Given that BPF can enter the bloodstream through the intestine to exert testicular toxicity, we further investigated its impact on intestinal metabolism in mice. Principal Component Analysis (PCA) revealed great differences in intestinal metabolites between the BPF-200 and control groups (Figure 2A,B). Volcano plot analysis identified 52 significantly upregulated and 32 downregulated metabolites (Figure 2C,D). Notably, histidine synthesis precursors (L-Histidinol) and histidine-related products (Carnosine) were markedly reduced following BPF exposure (Figure 2G,H). Concurrently, KEGG enrichment analysis demonstrated significant suppression of the histidine metabolic pathway in BPF-treated mice (Figure 2E,F). Subsequent ELISA validation confirmed that BPF exposure remarkably decreased histidine levels in mouse feces (Figure 2I). Further verification by qRT-PCR revealed differential downregulation in the mRNA expression levels of 8 key enzymes involved in the histidine metabolic pathway, collectively demonstrating that BPF disrupts intestinal histidine metabolism (Figure 3A–H).

### 3.3. BPF Induces GM Dysbiosis

**Figure 4:**
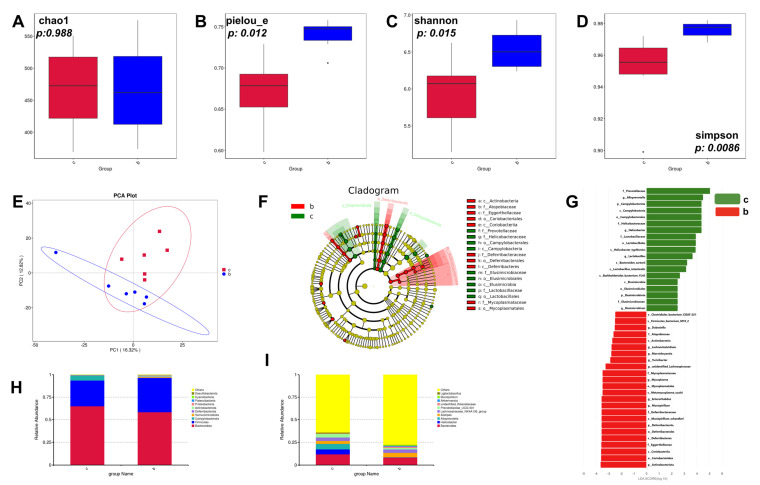
Gut microbiota dysbiosis of BPF mice. (**A**–**D**) The α-diversity including Chao, Pielou, Shannon and Simpson index, Control (red) and BPF-200 (blue); (**E**) PCA between the Control and BPF groups; (**F**,**G**) Statistics between the Control and BPF groups were identified using the line discriminant analysis (LDA) effect size (LEfSe) method. Cladogram illustrates the output of the LEfSe algorithm. Significantly distinct taxonomic nodes were colored, and the branch areas were shaded according to the effect size of the taxa (**F**). Taxa enriched in the Control (green) and BPF (red) groups with LDA score ≥ 5 were indicated (**G**); (**H**) The top ten bacteria with maximum abundance at the Phylum level; (**I**) The top ten bacteria with maximum abundance at the genus level. n = 6 per group; Wilcoxon test for α-diversity; PERMANOVA for PCA; LEfSe (Kruskal-Wallis + Wilcoxon).

Since histidine is metabolized by intestinal microbiota, next we performed shotgun metagenomic sequencing on the fecal samples of BPF-200 exposed and Control animals to assess the impact of GM on testicular injury. The α-diversity, assessed by the Chao1, Pielou_e and Simpson indices, exhibited significant differences between the two groups (Figure 4A–D). Meanwhile, using PCA based on Bray–Curtis distances, we found that the overall β-diversity of the GM composition was clearly distinct between the Control and BPF groups (Figure 4E). The results demonstrate that BPF exposure led to significant dysbiosis in mouse intestinal microbiota. To identify the key phylotypes that were prominently altered in the BPF group, we used the linear discriminant analysis (LDA) effect size (LEfSe) method to analyze the validated sequences at the genus and species levels (Figure 4F,G). At the phylum level, the abundance of Bacteroidota, Campylobacterota, Patescibacteria was decreased, while an increased abundance of Firmicutes, Verrucomicrobiota, Deferribacterota, Actinobacteriota, Proteobacteria, Cyanobacteria, and Desulfobacterota was observed in the BPF group (Figure 4H). The identification of the 10 most abundant species showed a decreased abundance of *Bacteroides*, *Helicobacter*, *Alloprevotella*, *Prevotellaceae_UCG-001*, *Ligilactobacillus* and an increased abundance of *Alistipes*, *Lachnospiraceae_NK4A136_group*, *Akkermansia*, *Mucispirillum* in the BPF group (Figure 4I). Metabolomic and GM analyses revealed that L-Histidinol and Carnosine exhibited strong correlations with Alloprevotella, which was reduced in the BPF group (Figure S1). Taken together, these results revealed the remarkable differences of the microbial community structure in mouse GMs after BPF-200 exposure, as well as a strong correlation between histidine-related metabolism and microbial differences.

### 3.4. BPF Exposure Is Associated with Reduced Testicular Histidine Levels and Altered LAT1/CD98-Related Markers

**Figure 5:**
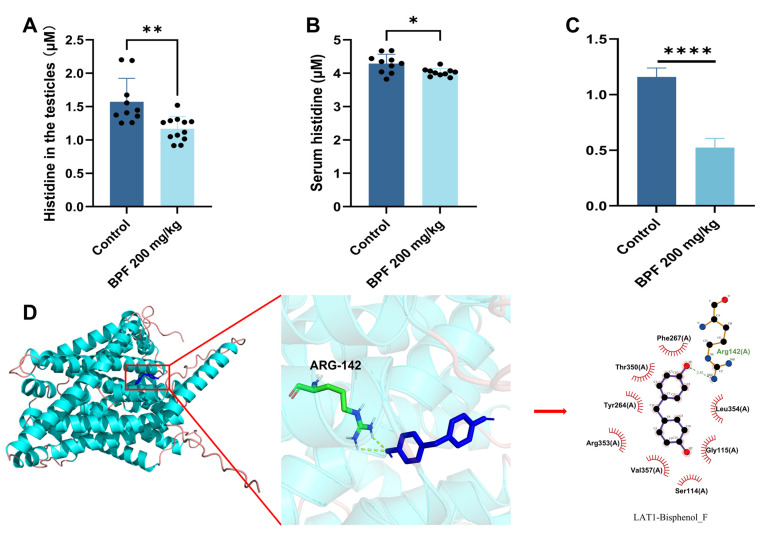
Association between BPF exposure and altered histidine availability and LAT1-related markers in testes. (**A**,**B**) ELISA analysis showing decreased histidine levels in the serum and testicular tissues of BPF-exposed mice; (**C**) qRT-PCR analysis demonstrating downregulated mRNA expression of LAT1 gene in mouse testicular tissues after BPF exposure; (**D**) Molecular docking predicting a potential interaction between BPF and LAT1. * *p* < 0.05, ** *p* < 0.01, **** *p* < 0.0001. Data are mean ± SEM; ANOVA/Tukey for (**A**,**B**); *t*-test for (**C**).

To explore whether altered histidine availability in the testes may be related to amino acid transport, we examined LAT1, a key component of the LAT1/CD98 heterodimeric amino acid transporter system. ELISA analysis showed decreased histidine levels in both serum and testicular tissues of BPF-exposed mice (Figure 5A,B). qRT-PCR analysis demonstrated reduced LAT1 mRNA expression in testicular tissues following BPF exposure (Figure 5C). Immunofluorescence analysis showed a similar decreasing trend in LAT1 signal in testicular tissues (Figure S2). In addition, molecular docking predicted a potential interaction between BPF and LAT1 (Figure 5D). Together, these results suggest that BPF exposure is associated with reduced testicular histidine availability and altered LAT1/CD98-related transport markers.

### 3.5. BPF Induces Ferroptosis in Testes

**Figure 6:**
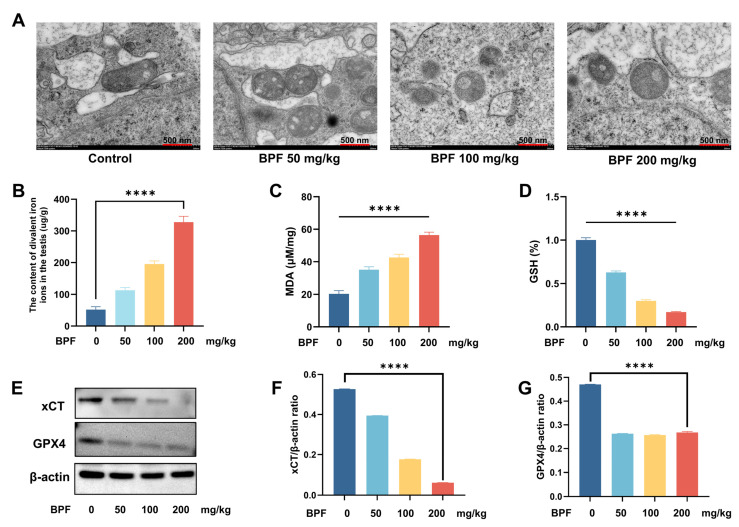
BPF exposure induces ferroptosis in mouse testicular tissues. (**A**) Transmission electron microscopy (TEM) reveals mitochondrial abnormalities, including cristae disintegration and mitochondrial swelling, in testicular tissues from the BPF-exposed mice; (**B**) Significant accumulation of Fe^2+^ in the testicular tissues after BPF exposure; (**C**,**D**) Elevated malondialdehyde (MDA) levels and decreased glutathione (GSH) content, indicating lipid peroxidation and antioxidant system impairment; (**E**) Western blot analysis shows downregulation of the cystine/glutamate antiporter (xCT) and glutathione peroxidase 4 (GPX4); (**F**,**G**) Bar graphs represent quantitative analysis for the corresponding protein bands in (**E**). **** *p* < 0.0001. Data are mean ± SEM. (**B**–**D**): n = 6 per group, ANOVA/Tukey. (**F**,**G**): n = 3 independent experiments, ANOVA/Tukey. (**A**): representative TEM from n = 3 per group, scale bars = 500 nm.

To further elucidate the mechanism underlying BPF-induced testicular damage via the histidine metabolic pathway, we postulated that BPF exposure might induce testicular ferroptosis because histidine is an endogenous ferrousion (Fe^2+^) chelator. Since Sertoli cells are the only somatic cell type supporting spermatogenic cells in the testicular seminiferous epithelium, we mainly observed their ultrastructural changes. The TEM examination revealed characteristic mitochondrial pathology, including cristae disintegration and mitochondrial swelling in these cells, which are all hallmark features of ferroptosis (Figure 6A). Subsequent biochemical analyses demonstrated a significant accumulation of Fe^2+^ in the BPF-exposed testicular tissues (Figure 6B), accompanied by elevated MDA levels and decreased GSH content, indicating lipid peroxidation and antioxidant system impairment (Figure 6C,D). Western blot analysis further confirmed the ferroptotic response, showing downregulation of the cystine/glutamate antiporter (xCT) and glutathione peroxidase 4 (GPX4) (Figure 6E–G). These findings collectively demonstrate that BPF exposure triggers ferroptosis in the testes, likely through dysregulation of the xCT system and subsequent disruption of cellular redox homeostasis.

### 3.6. BPF Triggers Ferroptosis in Cultured mSCs

**Figure 7:**
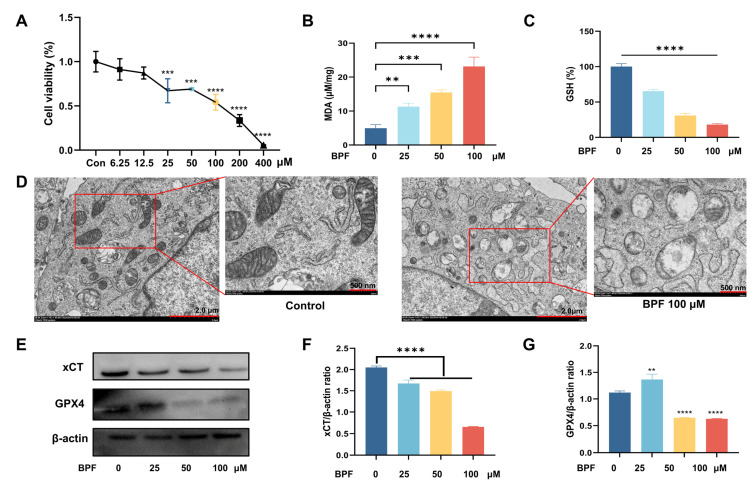
BPF induces ferroptosis in mouse Sertoli cells (mSCs) in vitro. (**A**) CCK-8 assay showing cell viability after BPF exposure at 25, 50, and 100 μM (resulting in ~50% mortality respectively); (**B**,**C**) Increased MDA and decreased GSH levels in BPF-treated cells; (**D**) TEM reveals mitochondrial damage including cristae disintegration and swelling; (**E**) Western blot shows downregulation of xCT and GPX4; (**F**,**G**) Bar graphs represent quantitative analysis for the corresponding protein bands in (**E**). ** *p* < 0.01, *** *p* < 0.001, **** *p* < 0.0001. Data are mean ± SEM. (**A**–**C**,**F**,**G**): n = 3 independent experiments (except (**A**) technical replicates n = 6); one-way ANOVA/Tukey. (**D**): representative TEM from three experiments, scale bars = 2 μm and 500 nm in magnified images.

**Figure 8:**
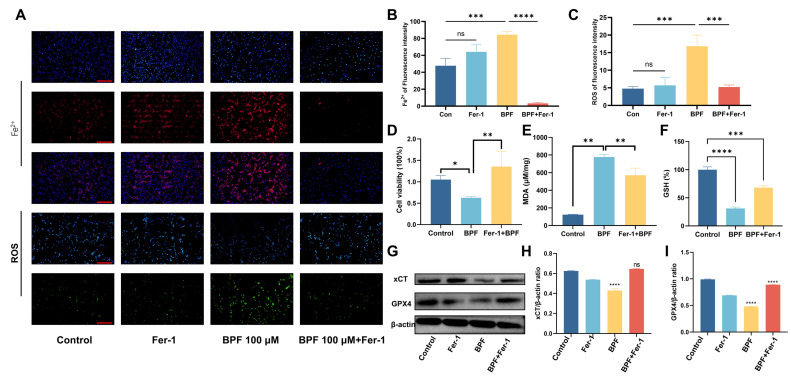
Ferrostatin-1 (Fer-1) inhibits BPF-100-induced ferroptosis in mSCs. (**A**) Fer-1 treatment (1 μM) reverses BPF-100-induced intracellular Fe^2+^ accumulation and attenuates ROS production. (**B**,**C**) Fluorescence intensity of Fe^2+^ and ROS, respectively; (**D**) Fer-1 ameliorates BPF-induced reduction in cell viability; (**E**,**F**) Fer-1 reverses MDA elevation and GSH depletion; (**G**) Western blot confirms that Fer-1 rescues xCT and GPX4 expression downregulated by BPF; (**H**,**I**) Bar graphs represent statistics for the corresponding protein bands in (**E**). ns = *p* > 0.05, * p < 0.05, ** *p* < 0.01, *** *p* < 0.001, **** *p* < 0.0001. Data are mean ± SEM. n = 3 independent experiments for (**B**,**C**,**E**,**F**,**H**,**I**); n = 6 technical replicates for (**D**). One-way ANOVA with Tukey’s post hoc test, scale bars = 200 μm.

Given that TEM revealed mitochondrial damage in in vivo Sertoli cells, and considering they are among the most critical somatic cells in male reproductive processes, we conducted in vitro experiments using mouse Sertoli cell line TM4 (mSCs). Initially, CCK-8 assays were performed to determine appropriate BPF concentrations, showing that BPF at 25, 50, and 100 μM concentrations resulted in approximately 50% cell mortality; therefore, these concentrations were selected for subsequent experiments (Figure 7A). Next, oxidative stress analysis showed that the MDA contents were significantly elevated, while the GSH levels were substantially reduced, both in a BPF dose-dependent manner (Figure 7B,C). Similar to that in in vivo Sertoli cells, BPF-100-exposed mSCs showed characteristic morphological damage, including mitochondrial cristae disintegration and swelling (Figure 7D). Western blot analysis also showed downregulation of anti-ferroptosis protein xCT and GPX4 (Figure 7F). To further validate these findings, we employed the ferroptosis inhibitor ferrostatin-1 (Fer-1) in cultured mSCs. Fe^2+^ fluorescence assays demonstrated that Fer-1 treatment (1 μM) reversed BPF-100 induced elevation of intracellular Fe^2+^ fluorescence intensity, and it also attenuated BPF-induced reactive oxygen species (ROS) accumulation simultaneously (Figure 8A–C). Moreover, Fer-1 ameliorated BPF-induced reduction in cell viability, reversed the increase in MDA levels, and restored the decline in GSH content (Figure 8D–F). Western blot analysis further confirmed that Fer-1 treatment rescued the BPF-induced downregulation of xCT and GPX4 expressions (Figure 8G–I). In addition, we performed Annexin V-FITC staining on TM4 cells, and the results showed that exposure to different concentrations of BPF did not induce apoptosis in the cells (Figure S3). Collectively, these results demonstrate that BPF triggers ferroptosis in mSCs by inhibiting the xC system, thereby compromising GPX4 production.

### 3.7. Histidine Supplementation Ameliorates BPF-Induced Ferroptosis in mSCs

**Figure 9:**
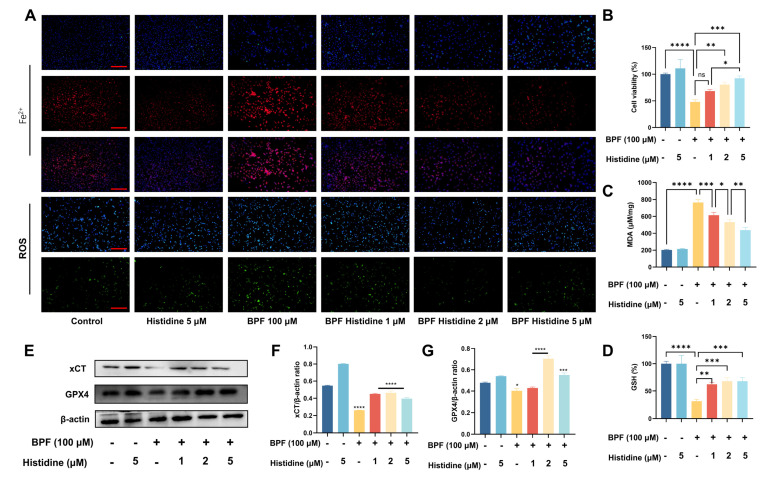
Exogenous histidine supplementation alleviates BPF-induced ferroptosis in mSCs. (**A**) Histidine (1, 2, 5 μM) reverses BPF-100-induced intracellular Fe^2+^ accumulation and attenuates ROS generation; (**B**) Histidine restores GSH levels; (**C**) Histidine reduces MDA content; (**D**) Histidine ameliorates BPF-induced reduction in cell viability; (**E**) Western blot shows histidine counteracts BPF-induced downregulation of xCT and GPX4. (**F**,**G**) Bar graphs represent quantitative results for the corresponding protein bands in (**E**). ns = *p* > 0.05, * *p* < 0.05, ** *p* < 0.01, *** *p* < 0.001, **** *p* < 0.0001. Data are mean ± SEM. n = 3 independent experiments.

To investigate the relationship between histidine metabolism and mSC ferroptosis, we supplemented exogenous histidine following BPF-100 exposure and evaluated the ferroptosis-related parameters. We observed that exogenous histidine greatly reversed BPF-induced elevation of ferrous ion levels in mSCs (Figure 9A, Figure S4). Concurrently, the increased MDA (Figure 9C) content and ROS accumulation induced by BPF were effectively alleviated (Figure 9A, Figure S4). The cell viability and GSH expression were also restored to the normal levels (Figure 9B,D). Notably, the downregulation of anti-ferroptosis proteins xCT and GPX4 was all counteracted by histidine supplementation (Figure 9E–G). These findings collectively indicate that exogenous histidine administration effectively mitigates BPF-induced ferroptosis in mSCs, scale bars = 200 μm.

### 3.8. Histidine Supplementation Ameliorates BPF-Induced Testicular Tissue Damage

**Figure 10:**
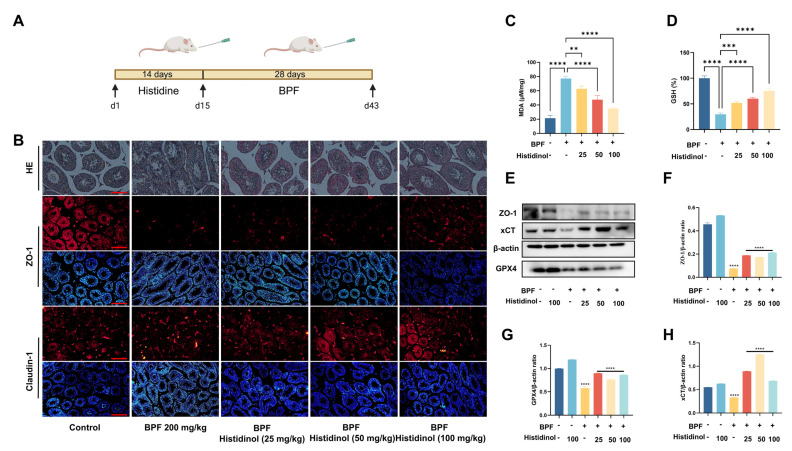
Exogenous histidine alleviates BPF-induced testicular damage. (**A**) Histidine repletion experimental procedure; (**B**) HE staining shows that histidine supplementation ameliorates BPF-induced seminiferous epithelium damage, including atrophy, loosening, and vacuolation; ZO-1 and Claudin-1 immunostaining indicates partial restoration of the BTB integrity; (**C**,**D**) Histidine reduces MDA content and increases GSH levels in mouse testes; (**E**) Western blot analysis demonstrates histidine reverses BPF-induced downregulation of ZO-1, xCT, and GPX4; (**F**–**H**) Bar graphs represent quantitative analysis for the corresponding protein bands in (**E**). ** *p* < 0.01, *** *p* < 0.001, **** *p* < 0.0001. Data are mean ± SEM. (**C**,**D**): n = 6 per group, ANOVA/Tukey. (**F**–**H**): n = 3 independent experiments, ANOVA/Tukey. (**B**): representative from n = 6 per group.

We further investigated whether supplementation with exogenous histidine in vivo could mitigate BPF-induced testicular tissue damage. The experimental procedure is shown in the figure (Figure 10A). HE staining demonstrated that, compared to the BPF-exposed group, histidine supplementation significantly alleviated BPF-caused damage to the seminiferous epithelium, including atrophy, loosening, and extensive vacuolation (Figure 10B, Figure S5A). Immunostaining of ZO-1 and Claudin-1 revealed that histidine supplementation partially restored the BTB integrity (Figure 10B, Figure S5B,C). Additionally, the MDA levels in testicular tissues were reduced, while the GSH levels were markedly increased (Figure 10C,D). Western blot analysis showed that exogenous histidine reversed the downregulation of xCT, GPX4, and ZO-1 induced by BPF exposure (Figure 10E–H). These findings collectively indicate that supplementation with exogenous histidine effectively ameliorates BPF-induced testicular tissue damage, scale bars = 200 μm.

## 4. Discussion

As an emerging environmental contaminant increasingly detected in human matrices, BPF represents a potential public health threat, particularly in the context of global declines in male reproductive health. While previous studies have documented its endocrine-disrupting properties, the precise molecular and metabolic pathways through which BPF exerts testicular toxicity remain elusive. Our study suggests this gap by identifying a previously unrecognized “GM-histidine-testis” axis as a potential central mechanism. The present study addresses this gap by proposing a previously underappreciated GM–histidine–testis axis as a possible contributor to BPF-induced testicular toxicity. Our data show that BPF exposure is accompanied by testicular injury, gut microbiota dysbiosis, disrupted intestinal histidine metabolism, reduced systemic and testicular histidine levels, altered LAT1-related markers, and ferroptosis-associated changes. These findings support, but do not definitively prove, a mechanistic link among gut microbiota disturbance, histidine deficiency, and testicular ferroptosis.

The testicular damage observed in this study—manifested as seminiferous tubule atrophy, epithelial vacuolization, and disrupted BTB integrity—aligns with established reproductive toxicity profiles of bisphenol analogues documented in prior literature [2,3]. The downregulation of key tight junction protein ZO-1 and Claudin-1 underscores the ability of BPF to disrupt the BTB, a structure vital for maintaining the immunoprivileged microenvironment necessary for spermatogenesis [11]. This structural impairment likely contributes to the spermatogenic defects observed, mirroring findings from models of cadmium and other environmental toxicant exposures [12]. Meanwhile, studies have reported that BPF at varying concentrations can induce diverse organ damage: concentrations ranging from 1 nM to 100 μM elicit neurotoxicity in rat neural stem cells [13]; exposure to 30 mg/kg BPF causes sexual dysfunction in rats [14,15,16]. Notably, research has demonstrated that BPF exposure at 200 mg/kg/day for 28 days can induce intestinal injury in rats [17]. This dosage aligns with the concentration employed in our study and further supports the possibility that BPF may exert its effects via intestinal pathways at this exposure level.

A novel aspect of our study is the identification of intestinal histidine metabolism as a primary target of BPF. The significant reduction in fecal histidine levels, histidine precursors, and related metabolites, coupled with the suppressed expression of key histidine metabolic enzymes, points to a profound disruption of this essential pathway. Given that GM play a crucial role in amino acid metabolism [18], the concomitant dysbiosis induced by BPF is likely a major contributor to this metabolic alteration. Our metagenomic analysis revealed a shift in microbial community structure, with decreased abundance of beneficial taxa (like *Alloprevotella*, *Bacteroides,* and *Ligilactobacillus*) and an increase in potential pathobionts (such as *Akkermansia* and *Mucispirillum*). This dysbiotic profile is reminiscent of changes linked to inflammation and metabolic disease [19], suggesting that BPF-induced microbial shifts may create a systemic pro-inflammatory state that exacerbates testicular damage. The concept of the “gut-testis axis” is gaining traction, with studies showing that GM-derived metabolites, such as short-chain fatty acids and tryptophan derivatives, can influence distal organ function, including reproduction [20,21]. Our results posit histidine or its deficiency as a new key player in this cross-talk.

The ferroptosis-related alterations observed in this study provide a plausible explanation for BPF-induced testicular injury. Histidine is a known chelator of ferrous iron (Fe^2+^) [22], and reduced histidine availability may therefore increase cellular susceptibility to iron-dependent lipid peroxidation. In the present study, BPF exposure was accompanied by decreased histidine levels, Fe^2+^ accumulation, elevated MDA, GSH depletion, and downregulation of the xCT/GPX4 antioxidant system. These changes are consistent with ferroptosis-related injury, as mitochondrial abnormalities, iron accumulation, lipid peroxidation, GSH/GPX4 depletion, and xCT downregulation are recognized features of ferroptosis [23,24]. Ferroptosis has also been implicated in stress- or toxicant-induced cellular injury, including heat stress-induced ferroptosis in Sertoli cells and cadmium- or arsenic-associated ferroptotic injury in other biological contexts [25,26,27]. In addition to reduced systemic histidine levels, our data suggest that testicular histidine availability may also be associated with changes in amino acid transport-related markers. LAT1, as part of the LAT1/CD98 heterodimeric amino acid transporter system, participates in the transport of large neutral amino acids, including histidine. In this study, BPF exposure was associated with reduced LAT1 mRNA expression and decreased LAT1 immunofluorescence signal in testicular tissues, and molecular docking predicted a possible interaction between BPF and LAT1. These findings raise the possibility that LAT1/CD98-related transport may be altered after BPF exposure. However, because direct LAT1/CD98 protein-level functional validation, transporter activity assays, and histidine uptake assays were not performed, we cannot conclude that BPF directly inhibits LAT1/CD98-mediated histidine transport. Therefore, the LAT1/CD98 pathway should be interpreted as a plausible but incompletely validated mechanism that may contribute to reduced testicular histidine availability. Sertoli cells are particularly important for spermatogenesis because they maintain BTB integrity and provide nutritional and structural support for developing germ cells [28]. Thus, reduced histidine availability, together with ferroptosis-related oxidative injury, may contribute to Sertoli cell dysfunction and subsequent impairment of the testicular microenvironment. Nevertheless, because histidine depletion alone was not experimentally induced in the absence of BPF, our data do not establish that histidine deficiency by itself is sufficient to trigger ferroptosis. Rather, histidine deficiency should be considered a potential contributing factor that may cooperate with other BPF-induced cellular stresses to promote ferroptosis-associated testicular damage.

The link between histidine deficiency and ferroptosis provides a coherent mechanism for BPF toxicity. This is supported by our rescue experiments, where exogenous histidine supplementation effectively mitigated all ferroptotic hallmarks, restored BTB integrity, and improved testicular morphology.

Our in vitro findings demonstrate that the specific ferroptosis inhibitor Fer-1 almost completely abrogated the ferroptotic cascade induced by BPF in mSCs. The primary mechanism of Fer-1 is the scavenging of lipid radicals, thereby halting the propagation of lipid peroxidation—the terminal step in ferroptosis [29]. Therefore, the protective effect of Fer-1 suggests that lipid peroxidation and ferroptosis-related processes contribute substantially to BPF-induced Sertoli cell injury. However, these findings do not exclude the involvement of additional stress responses or cell-death pathways, nor do they establish ferroptosis as the sole or earliest event in BPF toxicity. This interpretation is consistent with evidence that Sertoli cells may be vulnerable to environmental toxicant-induced ferroptosis due to their high metabolic activity and their role in maintaining redox homeostasis within the seminiferous epithelium [25]. The protection of Sertoli cell viability and function by Fer-1 directly explains the subsequent preservation of BTB integrity, as these cells are the principal architects of this barrier [28].

Building on this, the in vitro histidine supplementation elegantly bridged the gap between disrupted systemic histidine metabolism and testicular ferroptosis. The finding that exogenous histidine could mitigate BPF-induced ferroptosis in mSCs in a dose-dependent manner is pivotal. It positions histidine as an active, protective metabolite within the gut-testis axis. Histidine’s role as a potent chelator of Fe^2+^ is well-documented [22]. By supplementing histidine, we effectively restored the intracellular capacity to sequester labile iron, thereby preventing the iron-catalyzed Fenton reaction that generates the hydroxyl radicals responsible for initiating lipid peroxidation [30]. The observed restoration of GSH levels and GPX4 expression upon histidine treatment can be attributed to the interconnected nature of cellular antioxidant systems. By reducing the iron-driven oxidative burden, histidine may have conserved cellular reducing equivalents, allowing for the sustained reduction of GSH and the efficient function of GPX4. Furthermore, some studies suggest that histidine itself or its metabolites, such as carnosine, possess intrinsic antioxidant properties, capable of scavenging reactive carbonyl species and enhancing overall cellular resilience to oxidative stress [31]. However, rescue by exogenous histidine does not prove that histidine depletion alone initiates ferroptosis, because histidine supplementation may also affect oxidative stress, iron handling, or other metabolic pathways. Future studies using histidine-free culture conditions, dietary histidine restriction, or controlled histidine repletion in the absence of BPF are needed to determine whether histidine depletion is sufficient and/or necessary for ferroptosis induction.

The translational significance of these in vitro findings was powerfully confirmed by our in vivo rescue study. Administering histidine to BPF-exposed mice significantly ameliorated testicular histopathology, restored the expression and localization of BTB proteins (ZO-1 and Claudin-1), and reversed the testicular ferroptotic signature. This demonstrates that the deleterious effects of BPF along the gut-testis axis are not irreversible and can be therapeutically targeted. The in vivo efficacy of histidine likely operates through a dual mechanism: first, by directly supplementing the systemic and testicular histidine pool to chelate iron and inhibit ferroptosis locally, as seen in our in vitro data; and second, by potentially modulating the GM. While not directly tested in our rescue protocol, it is plausible that oral histidine could influence the GM community, possibly favoring the recovery of histidine-metabolizing or beneficial taxa, which in turn could contribute to a more favorable systemic metabolic and inflammatory state [20]. The restoration of BTB integrity is particularly critical. Since Sertoli cells are the cornerstone of the BTB, their rescue from ferroptosis by histidine directly prevents the breakdown of this vital structure, thereby preserving the immunoprivileged environment necessary for normal spermatogenesis [11]. This finding has profound implications, suggesting that nutritional interventions aimed at boosting histidine availability could protect against the reproductive toxicity of not only BPF but potentially other environmental insults that converge on the pathway of ferroptosis.

Our findings carry dual implications for environmental health. First, they provide a novel mechanistic basis for the risk assessment of BPF and structurally similar substitutes, highlighting the need to evaluate chemicals not only for hormonal activity but also for their potential to disrupt gut-organ axes and induce non-apoptotic cell death. Second, the efficacy of histidine supplementation in mitigating both the metabolic disturbance (histidine depletion) and the pathological endpoint (ferroptosis and BTB damage) points to a potential nutritional intervention strategy. While more research is needed to define optimal approaches, modulating histidine availability or targeting the gut microbiota could represent feasible avenues to reduce susceptibility to environmental reproductive toxicants. As BPF is increasingly used as a BPA substitute, its potential to cause similar or even novel toxicities cannot be overlooked [2,4]. Targeting the GM through probiotics or prebiotics to restore histidine-producing communities, or direct histidine supplementation, could represent promising therapeutic avenues to mitigate BPF-associated reproductive toxicity. This approach is supported by studies showing that nutritional interventions, such as omega-3 fatty acids, can protect against BPF-induced testicular dysfunction [32].

The BPF doses used (50–200 mg/kg/day) are higher than typical human environmental exposure (µg/kg range). This dose selection is consistent with previous BPF toxicology studies [17]. Higher doses were necessary to capture robust dose-dependent and multi-omics signals within the 28-day exposure period. Using body surface area conversion, the human equivalent of 200 mg/kg in mice is ~16 mg/kg/day, serving as a hazard identification benchmark. We acknowledge this as a mechanistic study, and future low-dose chronic studies are needed to confirm environmental relevance. We acknowledge that our in vitro BPF concentrations (25–100 μM) exceed environmental levels. These concentrations were selected based on CCK-8 assays to produce ~50% cytotoxicity and robust ferroptosis markers within 24 h, which is standard for mechanistic hazard identification. We recognize that nominal concentrations do not directly reflect in vivo tissue levels. Future low-dose, long-term studies are needed. This limitation has been noted. The reduced body weight gain at higher BPF doses suggests systemic toxicity. However, testicular injury was already significant at 50 mg/kg/day (minimal body weight effect), and in vitro direct BPF exposure recapitulated ferroptosis, sup-porting a testis-specific mechanism. While high-dose systemic effects could theoretically contribute, our pathway-specific rescue (histidine) argues against non-specific toxicity. Future low-dose chronic studies are needed to assess environmental relevance. The non-significant trend in testicular index likely reflects limited statistical power (Cohen’s d ≈ 1.4, power ≈ 50–60% with n = 6), which does not alter the main conclusions.

A limitation of the present study is the absence of a histidine-alone control group (i.e., mice receiving exogenous histidine supplementation without BPF exposure). While existing literature suggests that histidine at the doses used here does not exert discernible adverse effects on testicular structure or function, we cannot formally exclude the possibility that histidine itself influences the assessed endpoints. Future studies incorporating a histidine-alone control group would be valuable to fully dissociate the protective effects of histidine from potential baseline modulatory actions on the male reproductive system. A limitation of this study is that the observed associations between gut microbiota changes and testicular fer-roptosis do not prove causality. Future experiments using fecal microbiota transplantation (FMT) or germ-free mouse models are needed to establish a direct causal role of GM dysbiosis in BPF-induced testicular injury. Accordingly, we have tempered our causal claims throughout the manuscript. We further note that Annexin V-FITC staining con-firmed the absence of apoptosis in BPF-treated TM4 cells, supporting ferroptosis. Addi-tional markers (ACSL4, PTGS2) were not analyzed (limitation). Regarding cell type, our data indicate ferroptosis primarily in Sertoli cells; germ cells were not directly assessed. Future studies should address these points. We also acknowledge that we have not di-rectly tested whether histidine depletion alone is sufficient to induce ferroptosis. Direct deprivation experiments (e.g., histidine-free culture or dietary restriction) are needed to es-tablish causality, and we have noted this as a future direction.

Several questions remain for future investigation. First, the specific GM taxa whose shifts are most responsible for the histidine metabolic deficit need to be identified through functional metagenomics or gnotobiotic mouse models. Second, the potential crosstalk between ferroptosis and other cell death pathways, such as apoptosis or pyroptosis, which are also known to be activated by environmental stressors [5], should be explored. Third, while our docking study suggests an interaction, direct evidence that BPF binds to and inhibits LAT1 function in vitro is needed. Finally, the long-term effects of lower, more environmentally relevant doses of BPF on the gut-testis axis warrant thorough investigation.

## 5. Conclusions

**Figure 11:**
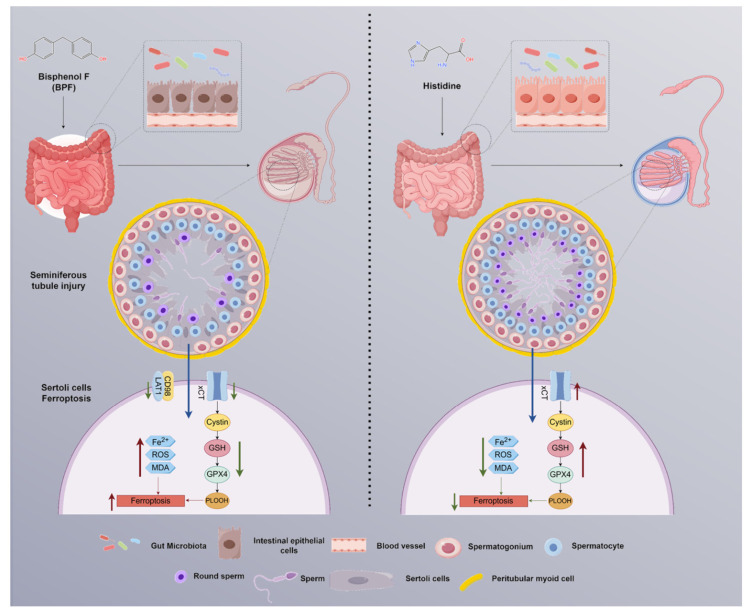
Schematic diagram of BPF-induced testicular injury in mice via the GM-testis axis. BPF disrupts the GM and histidine metabolism, subsequently enters the testicular tissue, and induces testicular ferroptosis through inhibition of the xC system. Histidine supplement reverses BPF-induced testicular injury.

In conclusion, this study suggests a novel pathogenic pathway whereby BPF exposure disrupts the GM, leading to impaired intestinal histidine metabolism, which may lead to histidine deficiency in the testes. This deficiency may promote ferroptosis in Sertoli cells, ultimately resulting in BTB dysfunction and testicular damage. These findings significantly advance our understanding of the reproductive toxicity of EDCs like bisphenol analogs and highlight the gut-testis axis and ferroptosis as critical targets for future research and intervention (Figure 11).

## Data Availability

The 16S rRNA sequencing data have been deposited in the NCBI SRA under the accession number PRJNA1433048. Untargeted metabolomics data (MTBLS13997): https://www.ebi.ac.uk/metabolights/reviewer269c07da-3658-4dd7-92d1-b00dff129712 (accessed on 14 July 2023).

## Supplementary Materials

The following supporting information can be downloaded at: https://www.mdpi.com/article/10.3390/antiox15060714/s1.

## Abbreviations

The following abbreviations are used in this manuscript:

BCA	Bicinchoninic acid
BPF	Bisphenol F
BPA	Bisphenol A
BTB	Blood-testis barrier
Fer-1	Ferrostatin-1
GM	Gut microbiota
GPX4	Glutathione peroxidase 4
HIS	L-histidine
LAT1	L-type amino acid transporter 1
xCT	Cystine/glutamate antiporter (SLC7A11)
ZO-1	Zonula occludens-1
