# Supplementary material for: Gut Microbiota-Mediated Histidine Deficiency Drives Testicular Ferroptosis Induced by Bisphenol F Exposure
Source: Antioxidants (Basel). 2026 Jun 4;15(6):714. doi: 10.3390/antiox15060714 (PMC13296130; doi:10.3390/antiox15060714)
Supplement: Supplementary file 1 [file antioxidants-15-00714-s001.zip › antioxidants-4295633-supplementary.pdf]

Supplementary Materials:

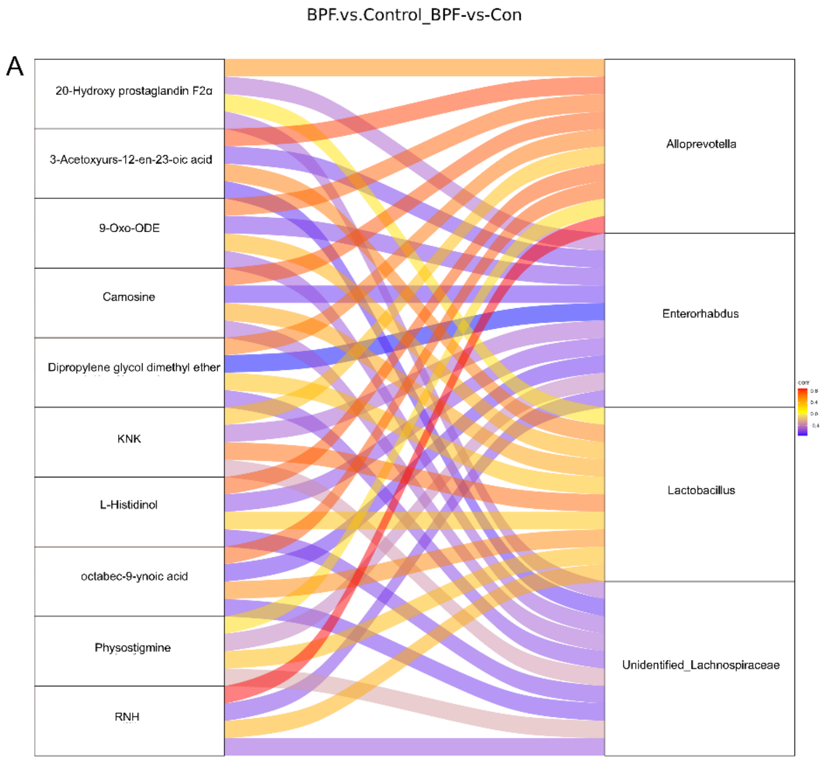

Figure S1. Combined Analysis of Metabolomics and Gut Microbiota. (A) Histidinol and Carnosine exhibited strong correlations with Alloprevotella.

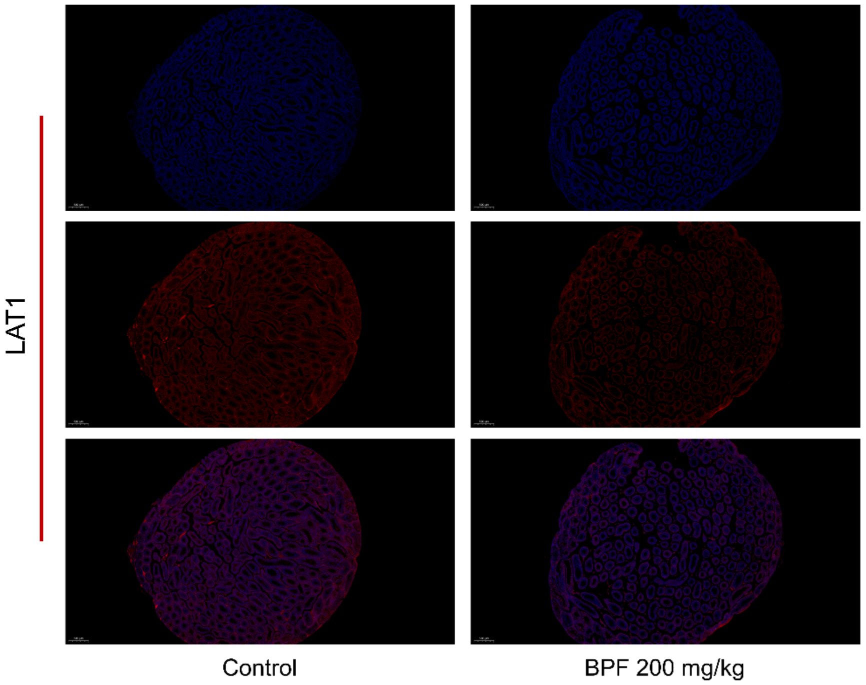

Figure S2. Immunofluorescence detection of LAT1 protein levels in testicular tissue.

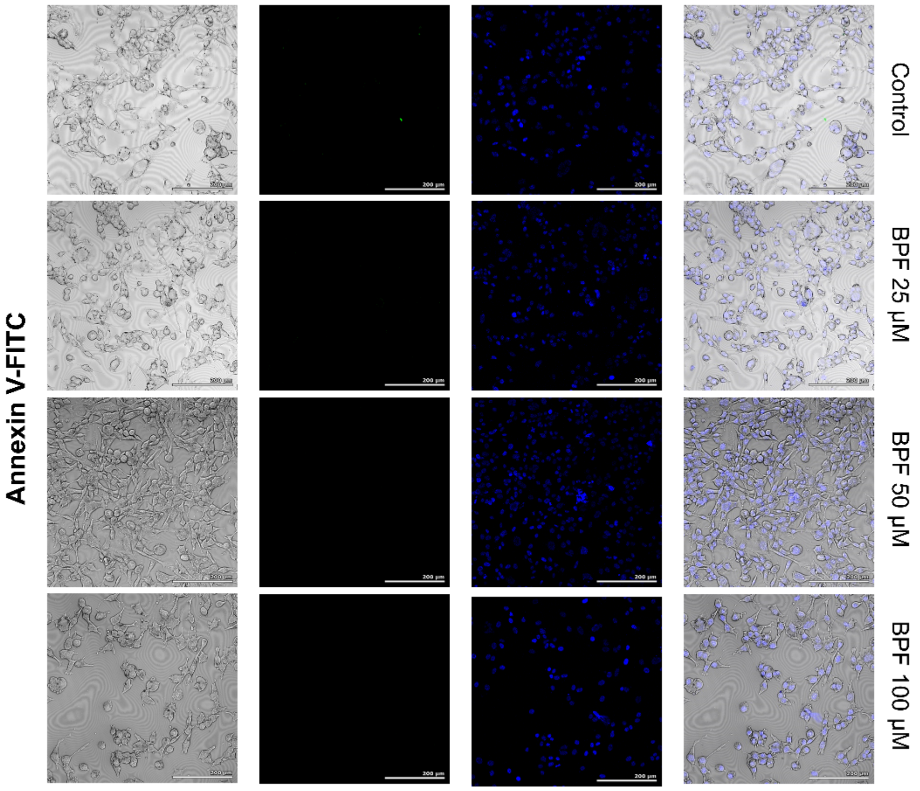

Figure S3. Annexin V-FITC detection of TM4 cell apoptosis.

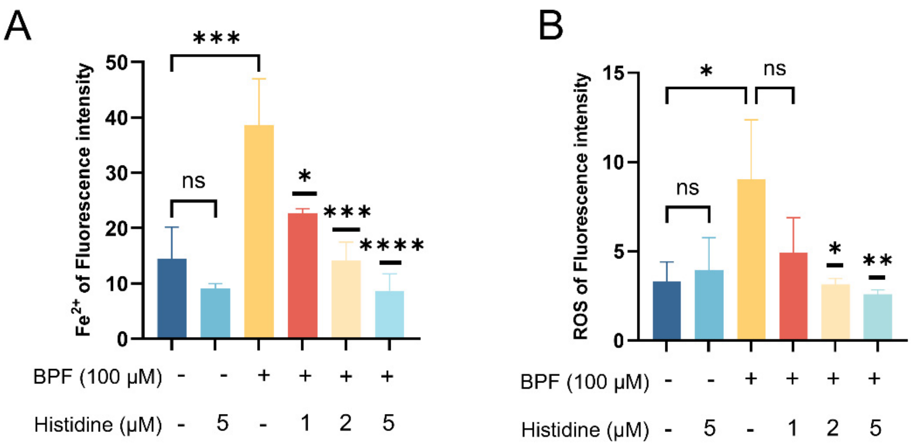

Figure S4. Fluorescence intensities of Fe2+ and ROS. (A) Fe2+ of fluorescence intensity; (B) ROS of fluorescence intensity. **P < 0.05, ***P < 0.001, ****P < 0.0001*

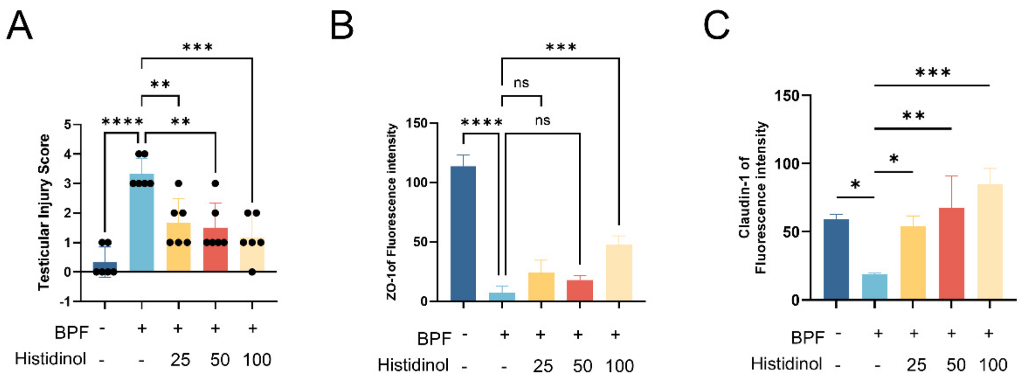

Figure S5. Evaluation of testicular damage and blood-testis barrier integrity. (A) Histopathological score of testicular tissue; (B) ZO-1 of fluorescence intensity; (C) Claudin-1 of fluorescence intensity. ***P < 0.05, ** P < 0.01, ***P < 0.001, ****P < 0.0001***

Table S1. Primers used for qRT-PCR.

Gene	Sequence
16s	F: 5' - AAGACCAAAGAGGGGGACCT-3'
	R: 5'- TGTCTCAGTTCCAGTGTGGC-3'
hisA	F: 5'- GTGGAAACCTATCTGCCCGT-3'
	R: 5'- ATCTGGCGCACACTTCTTCA-3'
hisB	F:5'- ATTGACGTTCAGGTGTGGCT-3'
	R: 5'- AGGTCGCCTTTGACGTTGAT-3'
hisC	F: 5'- GCTGAACGCCAACGAATACC-3'
	R: 5'- GCGCGAATCAGCAGTTCAAT-3'
hisD	F: 5'- ACCAACCACGTTCTACCGAC-3'
	R: 5'- TAACGGCATTTTTGTGGGCG-3'
hisF	F: 5'- ACATTCCGTTTTGTGTGGCG-3'
	R: 5'- GACCACAATACACTGCACGC-3'
hisG	F: 5'- ACTTTACCCTGCGTCGTCTG-3'
	R: 5'- TAACGCTTGAGCAGGTGAGG-3'
hisH	F: 5'- TGATCCTTGATACCGGCTGC-3'
	R: 5'- ACGGCTGACTTTGGGTTCAT-3'
hisI	F: 5'- ATGACACGCGTTCAATTTAAACAC-3'
	R: 5'- CTAGTCAGGATGATGGTGATGATGG-3'
LAT1	F: 5'- CCTCCAGCATGTAGGCGTAG-3'
	R: 5'- CATCATCGGCTCTGGCATCT-3
